# CA3 Synaptic Silencing Attenuates Kainic Acid-Induced Seizures and Hippocampal Network Oscillations^1^,^2^,^3^

**DOI:** 10.1523/ENEURO.0003-16.2016

**Published:** 2016-03-07

**Authors:** Lily M. Y. Yu, Denis Polygalov, Marie E. Wintzer, Ming-Ching Chiang, Thomas J. McHugh

**Affiliations:** Laboratory for Circuit and Behavioral Physiology, RIKEN Brain Science Institute, Wakoshi, Saitama, Japan 351-0198

**Keywords:** CA3, gamma, hippocampus, kainic acid, seizure, tetanus toxin

## Abstract

Epilepsy is a neurological disorder defined by the presence of seizure activity, manifest both behaviorally and as abnormal activity in neuronal networks. An established model to study the disorder in rodents is the systemic injection of kainic acid, an excitatory neurotoxin that at low doses quickly induces behavioral and electrophysiological seizures. Although the CA3 region of the hippocampus has been suggested to be crucial for kainic acid-induced seizure, because of its strong expression of kainate glutamate receptors and its high degree of recurrent connectivity, the precise role of excitatory transmission in CA3 in the generation of seizure and the accompanying increase in neuronal oscillations remains largely untested. Here we use transgenic mice in which CA3 pyramidal cell synaptic transmission can be inducibly silenced in the adult to demonstrate CA3 excitatory output is required for both the generation of epileptiform oscillatory activity and the progression of behavioral seizures.

## Significance Statement

The prevalence of epilepsy as a clinical concern has inspired the investigation of the mechanisms of seizure generation in animal models. A common model in the mouse is the injection of the neurotoxin kainic acid (KA). However, given the ability of KA to alter the activity of many cell types and circuits, it remains unclear how KA leads to seizure. Here we demonstrate that synaptic transmission from CA3 pyramidal cells in the hippocampus is necessary for KA-induced seizure activity, both behaviorally and physiologically. This establishes CA3 as the key locus for KA-induced pathophysiology and will aid in designing better models and interventions to understand and control seizures.

## Introduction

Temporal lobe epilepsy (TLE), a neurological disorder defined by the presences of severe seizures, remains a major clinical health issue worldwide. Animal models of TLE have been extremely useful in identifying the basic cellular mechanisms of epileptogenesis and have provided an assay system to test the effectiveness of interventions that may halt the onset or progression of seizures (Ben-Ari and Cossart, 2000; Leite et al., 2002). A particularly useful protocol employs the excitatory neurotoxin kainic acid (KA) to induce both acute seizures and in a subset of animals, progression to status epilepticus (Ben-Ari, 1985). Although strong evidence exists for a critical role of the CA3 region of the hippocampus in the induction of KA-seizures, because of its high expression of GluR6 receptors at the mossy fiber input synapses and strong recurrent connections, the precise circuit mechanisms of KA-induced pathological activity still are not fully elucidated (Lothman et al., 1991; Khalilov et al., 1999; Vincent and Mulle, 2009). Some of the uncertainty stems from the complex pattern of expression of multiple types of KA receptors on both excitatory and inhibitory neurons across the hippocampus (Carta et al., 2014), as well the ability of the drug to block glutamate transporters (Arriza et al., 1994) and some inward rectifying potassium current (Jabs et al., 1994; Melyan et al., 2002). Although it has been suggested that KA leads to excitation of both excitatory and inhibitory neurons with the balance ultimately tipping towards excitation and seizure generation, direct tests of this model have been lacking (Ben-Ari and Cossart, 2000).

In parallel to the induction of behavioral seizures, KA also induces strong increases in the amplitude of the neuronal oscillations in the hippocampus, particularly in the gamma (30–80 Hz) frequencies, that are thought to reflect the drug-induced hypersynchrony of the circuit (Traub et al., 2003). CA3 has been proposed as a likely origin of these gamma oscillations, as CA3 is involved in modulating conventional low-amplitude gamma oscillations during normal basal conditions (Bragin et al., 1995). However others have suggested that KA-induced gamma could arise from purely rhythmic inhibition or intrinsic properties of CA1 pyramidal cells (Whittington et al., 1995; Whittington et al., 2000; Craig and McBain, 2015). Further, mice lacking NMDA receptors specifically in CA3 pyramidal cells lacked KA-induced gamma oscillations, although these mice demonstrated increased sensitivity to KA-induced seizure, suggesting that a cause and effect relationship of these two phenomena may be a simplification of a more complex network interaction (Jinde et al., 2009).

Here we use triple transgenic mice in which the expression of the tetanus toxin (TeTX) light chain can be induced in CA3 pyramidal cells (CA3-TeTX mice) in the adult (Nakashiba et al., 2008) to address the necessity of CA3 pyramidal cell (PC) synaptic transmission in KA seizure generation. Our results demonstrate that CA3 PC transmission is necessary for KA-induced seizure activity, both behaviorally and physiologically. This establishes CA3 as a key locus for KA-induced pathophysiology and will aid in designing better models and interventions to understand and control seizures.

## Materials and Methods

### Animals

All the experiments were performed by operators blind to the animal’s genotype using male CA3-TeTX mice (triple-transgenic mice *KA1-Cre/+, TetO-TeTX/+*, *αCamKII-loxP-STOP-loxP-tTA/+*; background strain C57BL/6) between 16 and 36 weeks of age and their control male littermates (double-transgenic mice *KA1-Cre/+, TetO-TeTX/+*). TeTX expression was controlled via doxycycline (Dox) in the animals’ diet following established protocols (Nakashiba et al., 2008). We used Dox water (10 μg/ml, 1% sucrose) administered to the dams during the pregnancy and fostering periods and Dox food (10 mg/kg; Bioserve) following weaning to adulthood to keep TeTX in the repressed state. To induce TeTX expression in the adult the mice were switched to normal chow for a minimum of 21 d, a period that has been shown to lead to a complete blockade of CA3 synaptic transmission (Nakashiba et al., 2008). Following weaning two to four mice were housed per cage under the conditions of a 12 h light/dark cycle and *ad libitum* access to food and water. All experimental protocols were approved by the RIKEN Institutional Animal Care and Use Committee.

### Behavioral seizure scoring

All mice were switched to normal food to induce CA3 tetanus toxin expression in the mutants at least 21 d prior to the behavioral experiments. To induce seizure mice received an intraperitoneal injection of kainic acid (Sigma-Aldrich, K0250; 20 mg/kg in saline) and placed into a clean empty cage. Animals were monitored for 120 min following injection and seizure behavior was scored according to a modified Racine scale by an experimenter blind to the genotype (Racine, 1972): stage 0, normal behavior; stage 1, immobility and rigidity; stage 2, head bobbing; stage 3, forelimb clonus and rearing; stage 4, continuous rearing and falling; stage 5, clonic-tonic seizure; stage 6, death.

### c-fos immunohistochemistry

All mice were switched to normal food to induce CA3 tetanus toxin expression in the mutants at least 21 d prior to the *c-fos* induction experiments. Mice were administered an intraperitoneal injection of kainic acid (Sigma-Aldrich, K0250; 20 mg/kg in saline) or saline and 2.5–2.75 h later were transcardially perfused with 4% paraformaldehyde (PFA) in 0.1 m sodium phosphate buffer (PB). Brains were postfixed in 4% PFA and 50-μm-thick vibratome sections were prepared. After 3× 10 min PBS rinses, the sections were blocked in 3% normal donkey serum with 0.3% Triton-X in PBS for 2 h, and incubated overnight in primary antibody [rabbit α-c-fos, 1:2000; Cal Biochem, AB-5 (4-17) PC38] in blocking buffer. After 3× 10 min washes in PBS, the sections were incubated with AlexaFluor 594-conjugated donkey anti-rabbit (1:200) for 2 h at room temperature. Fluorescent images were collected (Leica DM6000B epifluorescent microscope with a Cy3 filter and a 5× objective) of sections containing the dorsal hippocampus (AP −1.58 mm to −1.94 mm from bregma) to quantify intensity of *c-fos* expression. Independent signal readings for the DG, CA3, and CA1 were calculated by measuring the cell body signal intensity (average of 5 randomly chosen areas, 50 × 50 square, A.U., in ImageJ), and the corresponding background signal (taken as dendritic region adjacent to cell body region, average of 3 areas, 50 × 50 square, A.U., in ImageJ). For each mouse, the normalized difference in signal intensity for each anatomical zone was calculated by dividing the difference between the average cell body signal and the average background signal divided by the sum of the two values.

### *In vivo* electrophysiology

Four groups of mice were used for *in vivo* recording, CA3-TeTX mutants (*n*=16) and littermate controls (*n*=4) off doxycycline for at least 21 d (OFF DOX) and CA3-TeTX mutants (*n*=6) and littermate controls (*n*=12) always on doxycycline containing food (ON DOX). No significant differences were observed in any parameter measured between the three control groups (controls ON DOX, controls OFF DOX, and CA3-TeTX ON DOX), thus they were combined to form a single control group. In all mice, the data was acquired using a 32-channel Digital Lynx 4SX acquisition system (Neuralynx). Mice were anaesthetized with urethane (1.3–1.5 g/kg), placed into a small animal stereotaxic frame and the skull exposed. Recording were conducted with a 32-channel single-shank silicon linear probe (NeuroNexus model no. A1-32-10mm-50-413-A32) lowered into the CA1 region of the right dorsal hippocampus (−1.9 mm bregma, 1.5 mm lateral) such that it spanned all layers of the structure. The signal was referenced to the most superficial cortical recording site and grounded to a skull screw. Proximity of the probe to the hippocampus was calculated by the depth of the probe via the micromanipulator and verified by the presence of complex spike bursts and occasional ripple oscillations. The site with maximum spiking activity was designated as the pyramidal cell layer and the anatomical location of all other sites was based on their relative spacing. Recording consisted of a baseline period (20 min) followed by a test period (110 min) that followed kainic acid injection (Sigma-Aldrich, K0250; 20 mg/kg in saline, i.p.). Local field potential (sampled at 32 kHz and filtered between 0.1 Hz and 6 kHz) and spike timestamps and waveforms (sampled at 32 kHz and filtered between 600 Hz and 6 kHz) were acquired on all 32 channels of the probe simultaneously.

### Data analyses

All analyses were conducted on the signal recorded in the stratum radiatum of CA1. First, the raw local field potentials were decimated 20 times, down to 1600 Hz by using custom software written in C. Power spectral density (PSD) of every LFP channel was calculated across the recording via the Welch's averaged modified periodogram method of spectral estimation (pwelch function, MATLAB, MathWorks). Parameters for the pwelch function were chosen as follows: windows size: 2048 samples (1.28 s), overlap size: 1024 samples (0.64 s), number of FFT points: 4096 (2.56 s). Resulting PSD matrices were used for calculating average traces of power spectral density changes over time within each frequency band of interest (theta: 3–8 Hz; slow gamma: 30–45 Hz; fast gamma: 55–80 Hz). Each trace was normalized by its mean value within the baseline recording period.

### Statistics

All analyses were conducted using GraphPad Prism v5.04. All time series data was compared between groups via a repeated-measure two-way ANOVA (genotype × time), following by *post hoc* testing at each time point via the Bonferroni correction. Student’s *t* test was used for pairwise comparisons. All values are reported as mean ± SEM. Statistical analyses are summarized in Table 1, superscript letters in the Results correspond to the rows in the table.

**Table 1.** Statistical table

	Data structure	Type of test	Observed power (α=0.05)
**a**	Normally distributed	Two-way repeated-measure ANOVA	<0.0001
		Bonferroni multiple comparisons (CA3-TeTX × control)	
		0	>0.05
		10	>0.05
		20	>0.05
		30	<0.01
		40	<0.0001
		50	<0.001
		60	<0.0001
		70	<0.0001
		80	<0.0001
		90	<0.0001
		100	<0.0001
		110	<0.0001
		120	<0.0001
**b**	Normally distributed	Unpaired *t* test	<0.0001
**c**	Normally distributed	Welch *t* test	0.0002
**d**	Normally distributed	Two-way ANOVA (genotype × drug)	0.003
		Bonferroni multiple comparisons (KA vs saline)	
		KA	<0.0001
		Saline	>0.05
**e**	Normally distributed	Two-way ANOVA (genotype × drug)	0.0011
		Bonferroni multiple comparisons (KA vs saline)	
		KA	<0.001
		Saline	>0.05
**f**	Normally distributed	Two-way ANOVA (genotype × drug)	0.0757
		Two-way repeated-measure ANOVA (genotype)	0.6035
		Two-way repeated-measure ANOVA (drug)	<0.0001
		Bonferroni multiple comparisons (KA vs saline)	
		KA	>0.05
		Saline	>0.05
**g**	Normally distributed	Two-way repeated-measure ANOVA (time × control group)	
		Theta (time × group)	0.9996
		Theta (time)	<0.0001
		Theta (group)	0.8227
		Low gamma (time × group)	0.7955
		Low gamma (time)	<0.0001
		Low gamma (group)	0.405
		High gamma (time × group)	0.9379
		High gamma (time)	<0.0001
		High gamma (group)	0.3334
**h**	Normally distributed	Two-way repeated-measure ANOVA (time × group) theta band	<0.0001
		Bonferroni multiple comparisons (CA3-TeTX × control ON DOX)	
		−20	*p*>0.05
		−15	*p*>0.05
		−10	*p*>0.05
		−5	*p*>0.05
		0	*p*>0.05
		5	*p*>0.05
		10	*p*>0.05
		15	*p*>0.05
		20	*p*>0.05
		25	*p*>0.05
		30	*p*>0.05
		35	*p*>0.05
		40	*p*>0.05
		45	*p*<0.05
		50	*p*<0.01
		55	*p*<0.001
		60	*p*<0.001
		65	*p*<0.001
		70	*p*<0.001
		75	*p*<0.0001
		80	*p*<0.0001
		85	*p*<0.0001
		90	*p*<0.0001
		95	*p*<0.0001
		100	*p*<0.0001
		105	*p*<0.0001
		Bonferroni multiple comparisons (CA3-TeTX × control OFF DOX)	
		−20	*p*>0.05
		−15	*p*>0.05
		−10	*p*>0.05
		−5	*p*>0.05
		0	*p*>0.05
		5	*p*>0.05
		10	*p*>0.05
		15	*p*>0.05
		20	*p*>0.05
		25	*p*>0.05
		30	*p*>0.05
		35	*p*<0.05
		40	*p*<0.05
		45	*p*<0.01
		50	*p*<0.05
		55	*p*<0.05
		60	*p*<0.01
		65	*p*<0.05
		70	*p*<0.05
		75	*p*>0.05
		80	*p*>0.05
		85	*p*>0.05
		90	*p*<0.01
		95	*p*<0.05
		100	*p*<0.01
		105	*p*<0.01
		Bonferroni multiple comparisons (CA3-TeTX × CA3-TeTX ON DOX)	
		−20	*p*>0.05
		−15	*p*>0.05
		−10	*p*>0.05
		−5	*p*>0.05
		0	*p*>0.05
		5	*p*>0.05
		10	*p*>0.05
		15	*p*>0.05
		20	*p*>0.05
		25	*p*>0.05
		30	*p*>0.05
		35	*p*>0.05
		40	*p*>0.05
		45	*p*>0.05
		50	*p*>0.05
		55	*p*>0.05
		60	*p*>0.05
		65	*p*>0.05
		70	*p*>0.05
		75	*p*<0.05
		80	*p*<0.01
		85	*p*<0.01
		90	*p*<0.01
		95	*p*<0.001
		100	*p*<0.001
		105	*p*<0.0001
		Bonferroni multiple comparisons (control ON DOX × control OFF DOX)	
		−20	*p*>0.05
		−15	*p*>0.05
		−10	*p*>0.05
		−5	*p*>0.05
		0	*p*>0.05
		5	*p*>0.05
		10	*p*>0.05
		15	*p*>0.05
		20	*p*>0.05
		25	*p*>0.05
		30	*p*>0.05
		35	*p*>0.05
		40	*p*>0.05
		45	*p*>0.05
		50	*p*>0.05
		55	*p*>0.05
		60	*p*>0.05
		65	*p*>0.05
		70	*p*>0.05
		75	*p*>0.05
		80	*p*>0.05
		85	*p*>0.05
		90	*p*>0.05
		95	*p*>0.05
		100	*p*>0.05
		105	*p*>0.05
		Bonferroni multiple comparisons (control ON DOX × CA3-TeTX ON DOX)	
		−20	*p*>0.05
		−15	*p*>0.05
		−10	*p*>0.05
		−5	*p*>0.05
		0	*p*>0.05
		5	*p*>0.05
		10	*p*>0.05
		15	*p*>0.05
		20	*p*>0.05
		25	*p*>0.05
		30	*p*>0.05
		35	*p*>0.05
		40	*p*>0.05
		45	*p*>0.05
		50	*p*>0.05
		55	*p*>0.05
		60	*p*>0.05
		65	*p*>0.05
		70	*p*>0.05
		75	*p*>0.05
		80	*p*>0.05
		85	*p*>0.05
		90	*p*>0.05
		95	*p*>0.05
		100	*p*>0.05
		105	*p*>0.05
		Bonferroni multiple comparisons (control OFF DOX × CA3-TeTX ON DOX)	
		−20	*p*>0.05
		−15	*p*>0.05
		−10	*p*>0.05
		−5	*p*>0.05
		0	*p*>0.05
		5	*p*>0.05
		10	*p*>0.05
		15	*p*>0.05
		20	*p*>0.05
		25	*p*>0.05
		30	*p*>0.05
		35	*p*>0.05
		40	*p*>0.05
		45	*p*>0.05
		50	*p*>0.05
		55	*p*>0.05
		60	*p*>0.05
		65	*p*>0.05
		70	*p*>0.05
		75	*p*>0.05
		80	*p*>0.05
		85	*p*>0.05
		90	*p*>0.05
		95	*p*>0.05
		100	*p*>0.05
		105	*p*>0.05
	Normally distributed	Two-way repeated-measure ANOVA (time × group) low gamma band	<0.0001
		Bonferroni multiple comparisons (CA3-TeTX × control ON DOX)	
		−20	*p*>0.05
		−15	*p*>0.05
		−10	*p*>0.05
		−5	*p*>0.05
		0	*p*>0.05
		5	*p*>0.05
		10	*p*>0.05
		15	*p*>0.05
		20	*p*>0.05
		25	*p*>0.05
		30	*p*>0.05
		35	*p*>0.05
		40	*p*<0.05
		45	*p*<0.01
		50	*p*<0.0001
		55	*p*<0.0001
		60	*p*<0.0001
		65	*p*<0.0001
		70	*p*<0.0001
		75	*p*<0.0001
		80	*p*<0.0001
		85	*p*<0.0001
		90	*p*<0.0001
		95	*p*<0.0001
		100	*p*<0.0001
		105	*p*<0.001
		Bonferroni multiple comparisons (CA3-TeTX × control OFF DOX)	
		−20	*p*>0.05
		−15	*p*>0.05
		−10	*p*>0.05
		−5	*p*>0.05
		0	*p*>0.05
		5	*p*>0.05
		10	*p*>0.05
		15	*p*>0.05
		20	*p*>0.05
		25	*p*>0.05
		30	*p*>0.05
		35	*p*>0.05
		40	*p*>0.05
		45	*p*>0.05
		50	*p*>0.05
		55	*p*>0.05
		60	*p*>0.05
		65	*p*>0.05
		70	*p*>0.05
		75	*p*>0.05
		80	*p*>0.05
		85	*p*>0.05
		90	*p*>0.05
		95	*p*>0.05
		100	*p*>0.05
		105	*p*>0.05
		Bonferroni multiple comparisons (CA3-TeTX × CA3-TeTX ON DOX)	
		−20	*p*>0.05
		−15	*p*>0.05
		−10	*p*>0.05
		−5	*p*>0.05
		0	*p*>0.05
		5	*p*>0.05
		10	*p*>0.05
		15	*p*>0.05
		20	*p*>0.05
		25	*p*>0.05
		30	*p*>0.05
		35	*p*>0.05
		40	*p*>0.05
		45	*p*>0.05
		50	*p*<0.05
		55	*p*>0.05
		60	*p*>0.05
		65	*p*>0.05
		70	*p*>0.05
		75	*p*>0.05
		80	*p*>0.05
		85	*p*>0.05
		90	*p*>0.05
		95	*p*>0.05
		100	*p*>0.05
		105	*p*>0.05
		Bonferroni multiple comparisons (control ON DOX × control OFF DOX)	
		−20	*p*>0.05
		−15	*p*>0.05
		−10	*p*>0.05
		−5	*p*>0.05
		0	*p*>0.05
		5	*p*>0.05
		10	*p*>0.05
		15	*p*>0.05
		20	*p*>0.05
		25	*p*>0.05
		30	*p*>0.05
		35	*p*>0.05
		40	*p*>0.05
		45	*p*>0.05
		50	*p*>0.05
		55	*p*>0.05
		60	*p*>0.05
		65	*p*>0.05
		70	*p*>0.05
		75	*p*>0.05
		80	*p*>0.05
		85	*p*>0.05
		90	*p*>0.05
		95	*p*>0.05
		100	*p*>0.05
		105	*p*>0.05
		Bonferroni multiple comparisons (control ON DOX × CA3-TeTX ON DOX)	
		−20	*p*>0.05
		−15	*p*>0.05
		−10	*p*>0.05
		−5	*p*>0.05
		0	*p*>0.05
		5	*p*>0.05
		10	*p*>0.05
		15	*p*>0.05
		20	*p*>0.05
		25	*p*>0.05
		30	*p*>0.05
		35	*p*>0.05
		40	*p*>0.05
		45	*p*>0.05
		50	*p*>0.05
		55	*p*>0.05
		60	*p*>0.05
		65	*p*>0.05
		70	*p*>0.05
		75	*p*>0.05
		80	*p*>0.05
		85	*p*>0.05
		90	*p*>0.05
		95	*p*>0.05
		100	*p*>0.05
		105	*p*>0.05
		Bonferroni multiple comparisons (control OFF DOX × CA3-TeTX ON DOX)	
		−20	*p*>0.05
		−15	*p*>0.05
		−10	*p*>0.05
		−5	*p*>0.05
		0	*p*>0.05
		5	*p*>0.05
		10	*p*>0.05
		15	*p*>0.05
		20	*p*>0.05
		25	*p*>0.05
		30	*p*>0.05
		35	*p*>0.05
		40	*p*>0.05
		45	*p*>0.05
		50	*p*>0.05
		55	*p*>0.05
		60	*p*>0.05
		65	*p*>0.05
		70	*p*>0.05
		75	*p*>0.05
		80	*p*>0.05
		85	*p*>0.05
		90	*p*>0.05
		95	*p*>0.05
		100	*p*>0.05
		105	*p*>0.05
	Normally distributed	Two-way repeated-measure ANOVA (time × group) high gamma band	<0.0001
		Bonferroni multiple comparisons (CA3-TeTX × control ON DOX)	
		−20	*p*>0.05
		−15	*p*>0.05
		−10	*p*>0.05
		−5	*p*>0.05
		0	*p*>0.05
		5	*p*>0.05
		10	*p*>0.05
		15	*p*>0.05
		20	*p*>0.05
		25	*p*>0.05
		30	*p*<0.001
		35	*p*<0.01
		40	*p*<0.001
		45	*p*<0.001
		50	*p*<0.001
		55	*p*<0.0001
		60	*p*<0.0001
		65	*p*<0.001
		70	*p*<0.001
		75	*p*<0.001
		80	*p*<0.01
		85	*p*<0.01
		90	*p*<0.01
		95	*p*<0.05
		100	*p*<0.05
		105	*p*<0.05
		Bonferroni multiple comparisons (CA3-TeTX × control OFF DOX)	
		−20	*p*>0.05
		−15	*p*>0.05
		−10	*p*>0.05
		−5	*p*>0.05
		0	*p*>0.05
		5	*p*>0.05
		10	*p*>0.05
		15	*p*>0.05
		20	*p*>0.05
		25	*p*>0.05
		30	*p*>0.05
		35	*p*>0.05
		40	*p*>0.05
		45	*p*>0.05
		50	*p*>0.05
		55	*p*>0.05
		60	*p*>0.05
		65	*p*>0.05
		70	*p*>0.05
		75	*p*>0.05
		80	*p*>0.05
		85	*p*>0.05
		90	*p*>0.05
		95	*p*>0.05
		100	*p*>0.05
		105	*p*>0.05
		Bonferroni multiple comparisons (CA3-TeTX × CA3-TeTX ON DOX)	
		−20	*p*>0.05
		−15	*p*>0.05
		−10	*p*>0.05
		−5	*p*>0.05
		0	*p*>0.05
		5	*p*>0.05
		10	*p*>0.05
		15	*p*>0.05
		20	*p*>0.05
		25	*p*>0.05
		30	*p*>0.05
		35	*p*>0.05
		40	*p*<0.01
		45	*p*<0.01
		50	*p*<0.01
		55	*p*>0.05
		60	*p*>0.05
		65	*p*>0.05
		70	*p*>0.05
		75	*p*>0.05
		80	*p*>0.05
		85	*p*>0.05
		90	*p*>0.05
		95	*p*>0.05
		100	*p*>0.05
		105	*p*>0.05
		Bonferroni multiple comparisons (control ON DOX × control OFF DOX)	
		−20	*p*>0.05
		−15	*p*>0.05
		−10	*p*>0.05
		−5	*p*>0.05
		0	*p*>0.05
		5	*p*>0.05
		10	*p*>0.05
		15	*p*>0.05
		20	*p*>0.05
		25	*p*>0.05
		30	*p*>0.05
		35	*p*>0.05
		40	*p*>0.05
		45	*p*>0.05
		50	*p*>0.05
		55	*p*>0.05
		60	*p*>0.05
		65	*p*>0.05
		70	*p*>0.05
		75	*p*>0.05
		80	*p*>0.05
		85	*p*>0.05
		90	*p*>0.05
		95	*p*>0.05
		100	*p*>0.05
		105	*p*>0.05
		Bonferroni multiple comparisons (control ON DOX × CA3-TeTX ON DOX)	
		−20	*p*>0.05
		−15	*p*>0.05
		−10	*p*>0.05
		−5	*p*>0.05
		0	*p*>0.05
		5	*p*>0.05
		10	*p*>0.05
		15	*p*>0.05
		20	*p*>0.05
		25	*p*>0.05
		30	*p*>0.05
		35	*p*>0.05
		40	*p*>0.05
		45	*p*>0.05
		50	*p*>0.05
		55	*p*>0.05
		60	*p*>0.05
		65	*p*>0.05
		70	*p*>0.05
		75	*p*>0.05
		80	*p*>0.05
		85	*p*>0.05
		90	*p*>0.05
		95	*p*>0.05
		100	*p*>0.05
		105	*p*>0.05
		Bonferroni multiple comparisons (control OFF DOX × CA3-TeTX ON DOX)	
		−20	*p*>0.05
		−15	*p*>0.05
		−10	*p*>0.05
		−5	*p*>0.05
		0	*p*>0.05
		5	*p*>0.05
		10	*p*>0.05
		15	*p*>0.05
		20	*p*>0.05
		25	*p*>0.05
		30	*p*>0.05
		35	*p*>0.05
		40	*p*>0.05
		45	*p*>0.05
		50	*p*>0.05
		55	*p*>0.05
		60	*p*>0.05
		65	*p*>0.05
		70	*p*>0.05
		75	*p*>0.05
		80	*p*>0.05
		85	*p*>0.05
		90	*p*>0.05
		95	*p*>0.05
		100	*p*>0.05
		105	*p*>0.05
**i**	Normally distributed	Two-way repeated-measure ANOVA (time × group)	<0.0001
		Bonferroni multiple comparisons (CA3-TeTX × control)	
		−20	>0.05
		−15	>0.05
		−10	>0.05
		−5	>0.05
		0	>0.05
		5	>0.05
		10	>0.05
		15	>0.05
		20	>0.05
		25	>0.05
		30	>0.05
		35	<0.05
		40	<0.01
		45	<0.0001
		50	<0.0001
		55	<0.0001
		60	<0.0001
		65	<0.0001
		70	<0.0001
		75	<0.0001
		80	<0.0001
		85	<0.0001
		90	<0.0001
		95	<0.0001
		100	<0.0001
		105	<0.0001
**j**	Normally distributed	Two-way repeated-measure ANOVA (time × group)	<0.0001
		Bonferroni multiple comparisons (CA3-TeTX × control)	
		−20	>0.05
		−15	>0.05
		−10	>0.05
		−5	>0.05
		0	>0.05
		5	>0.05
		10	>0.05
		15	>0.05
		20	<0.05
		25	<0.01
		30	<0.0001
		35	<0.0001
		40	<0.0001
		45	<0.0001
		50	<0.0001
		55	<0.0001
		60	<0.0001
		65	<0.0001
		70	<0.0001
		75	<0.0001
		80	<0.0001
		85	<0.001
**k**	Normally distributed	Two-way repeated-measure ANOVA (time × group)	<0.0001
		Bonferroni multiple comparisons (CA3-TeTX × control)	
		−20	>0.05
		−15	>0.05
		−10	>0.05
		−5	>0.05
		0	>0.05
		5	>0.05
		10	>0.05
		15	>0.05
		20	>0.05
		25	<0.05
		30	<0.01
		35	<0.001
		40	<0.0001
		45	<0.0001
		50	<0.0001
		55	<0.0001
		60	<0.001
		65	<0.001
		70	<0.01
		75	<0.01
		80	<0.01
		85	<0.01
		90	<0.01
		95	<0.01
		100	<0.01
		105	<0.05

## Results

**Figure 1.**
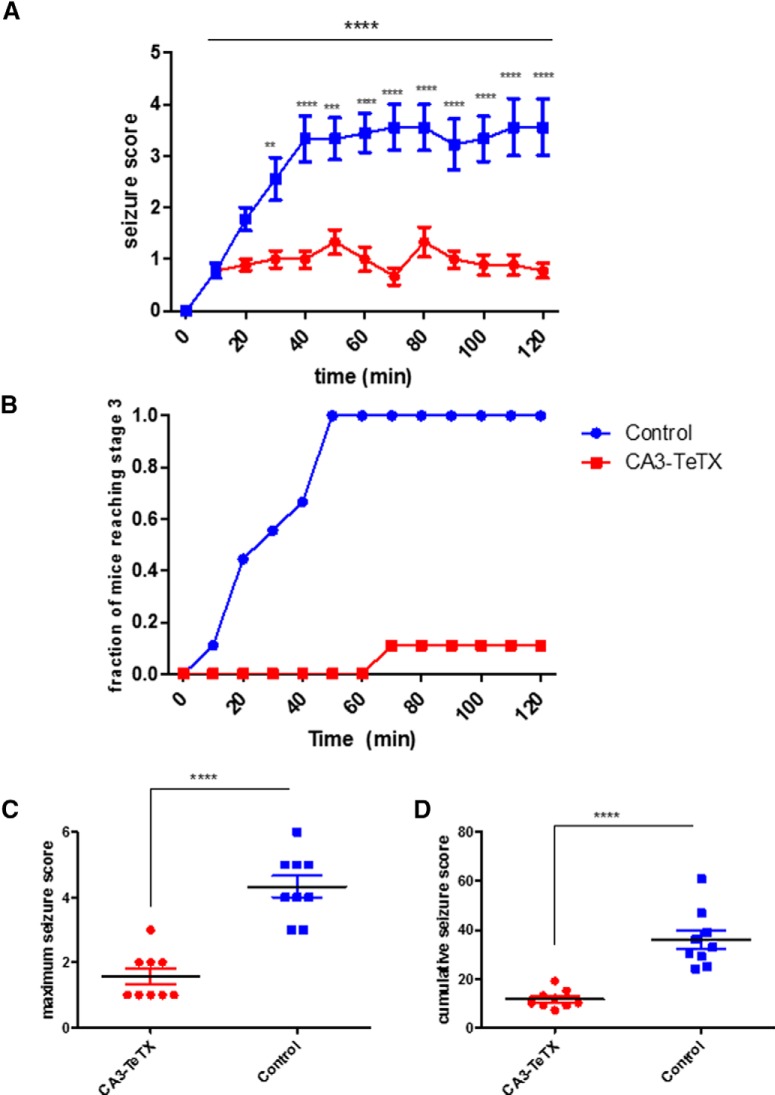
**Genetic blockade of CA3 synaptic transmission attenuates acute KA-induced seizures. *A***, The mean behavioral seizure score following kainic acid injection was significantly reduced in CA3-TeTX mice. Analysis revealed that (***B***) the fraction of CA3-TeTX mice reaching stage 3 seizures (forelimb clonus and rearing) was reduced compared with control mice. The mutant mice also had (***C***) a significantly lower maximal seizure score and (***D***) a lower cumulative seizure score than control mice. ****p<0.0001, ***p<0.001, **p<0.01. Control, *n*=9; CA3-TeTX *n*=9.

In CA3-TeTX mice the removal of Dox from the diet leads to expression of the tetanus toxin light-chain (TeTX) in CA3 pyramidal cells. TeTX efficiently cleaves the VAMP2 protein required for synaptic vesicle release, leading to a loss of synaptic transmission from CA3 without altering cellular excitability (Nakashiba et al., 2008). Using this model, we first asked whether CA3 PC output was required for the induction of acute behavioral seizure following systematic injection of KA. KA (20 mg/kg) was injected intraperitoneally into CA3-TeTX mice (*n*=9) and littermate controls (*n*=9) and behavioral seizure responses were scored using the modified Racine scale (Racine, 1972) for 120 min postinjection. We observed a significant difference in behavior between genotypes across the durations of the experiment^a^ [two-way repeated-measure ANOVA, *F*_(1,12)_ (genotype × time)=8.79, *p*<0.0001], with control mice showing significantly more severe seizures beginning 30 min after injection (Fig. 1*A*). This decrease in sensitivity to KA-induced seizure was also reflected in the fraction of mice reaching stage 3 (Fig. 1*B*; controls 9/9, CA3-TeTX 1/9), a decrease in the maximal seizure score^b^ (Fig. 1*C*; *t* test, *p*<0.0001) and a significant reduction in the cumulative seizure score in the CA3-TeTX mice^c^ (Fig. 1*D*; Welsh’s *t* test, *p*<0.0001).

**Figure 2.**
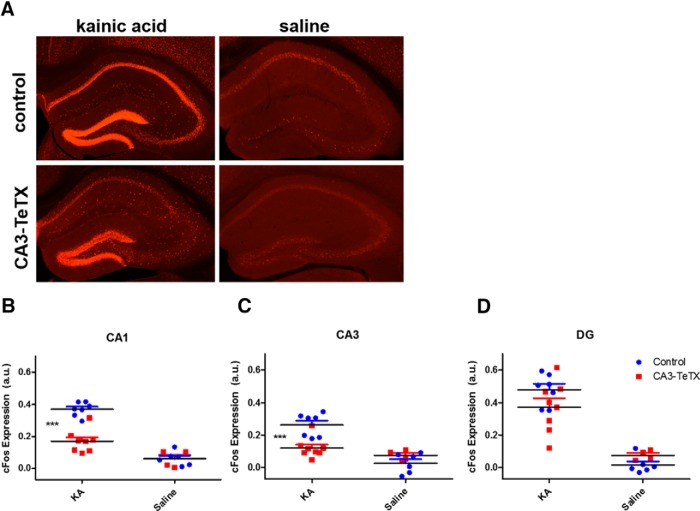
**Loss of CA3 output causes a decrease in *c-fos* expression in the CA3 and CA1 subfields following KA-induced seizure. *A***, Examples of *c-fos* protein expression in the dorsal hippocampus of control (top) and CA3-TeTX (bottom) mice 2.5 h after injection with kainic acid (left) or saline (right). Quantification of the fluorescent *c-fos* expression signal finds significantly higher signal in the (***B***) CA1 and (***C***) CA3 regions of the control mice injected with KA compared with the CA3-TeTX/KA and both saline groups. However, in the (***D***) DG, robust *c-fos* expression was evident in both genotypes following KA injection. ***p<0.001. Control + KA, *n*=7; control + saline, *n*=6; CA3-TeTX + KA, *n*=8; CA3-TeTX + saline, *n*=4.

Having established that a loss of CA3 synaptic transmission severely attenuates the behavioral response to KA injection, we next addressed how the individual subfields of the hippocampus responded following drug administration. CA3-TeTX and littermate controls were once again systemically injected with KA (20 mg/kg, i.p.), and 150–165 min later the mice were euthanized and the brains prepared for immunohistochemistry. To examine neuronal activation we compared *c-fos* protein expression, a reliable indicator of neuronal activity (Labiner et al., 1993), across each of three hippocampal subfields in four groups of mice: CA3-TeTX and control mice injected with KA or CA3-TeTX and control mice injected with saline. In CA1 we found a significant interaction between genotype and drug^d^ [two-way ANOVA, *F*_(1,21)_ (genotype × drug)=18.35, *p*=0.0003] and a significant decrease in *c-fos* expression in KA-injected CA3-TeTX mice compared with controls^d^ (Bonferroni *post hoc* test, *p*<0.0001) (Fig. 2*A*,*B*). A similar pattern of results was observed in CA3, where once again there was a significant interaction between genotype and drug^e^ [two-way ANOVA, *F*_(1,21)_ (genotype × drug)=14.39, *p*=0.0011] and a significant decrease in *c-fos* expression in KA-injected CA3-TeTX mice^e^ (Bonferroni *post hoc* test, *p*<0.0001) (Fig. 2*C*). In the DG however, a distinct pattern of results was observed. As expected, there was a significant effect of the drug^f^ [two-way ANOVA, *F*_(1,21)_ (drug)=72.96, *p*0.0001] but no significant effect of genotype^f^ (*F*=0.28, *p*=0.60) or interaction between genotype and drug^f^ (*F*=3.49, *p*=0.08) (Fig. 2*D*). These data indicate that although KA injection can directly activate neurons in the DG in both groups of mice, the progression of neuronal activity through the classic trisynaptic hippocampal circuit into CA1 requires CA3 pyramidal cell synaptic transmission.

Given the inability of KA to reliably induce behavioral seizure or neuronal activation in the CA fields of the CA3-TeTX mice we next addressed how CA3 synaptic silencing influences KA-induced oscillations in CA1. Four groups of mice were subject to *in vivo* electrophysiology following KA administration, CA3-TeTX mice OFF DOX (mutant, *n*=16) and three control groups (control mice ON DOX, *n*=12; control mice OFF DOX, *n*=4; CA3-TeTX mice ON DOX, *n*=6). The mice were anesthetized with urethane and acute recordings of hippocampal local field potential were made using a multisite silicon probe spanning CA1 from stratum oriens to stratum lacunosum-moleculare. Following a 20 min baseline recording the mice received a single injection of KA (20 mg/kg, i.p.) and recording continued for 110 min. Based on previous work (Fisahn et al., 2004; Sakatani et al., 2007; Jinde et al., 2009), we focused on the amplitude of three common hippocampal oscillations, theta (3–8 Hz) and slow (30–45 Hz) and fast (55–80 Hz) gamma, recorded in the str. radiatum, which contains the Schaffer collateral projections from CA3 to CA1. We observed no differences in the power of the any of the frequency bands between the three types of control mice at any point before or after drug administration^g^. Further, when we compared the power of the any of the frequency bands between all four groups of mice, a two-way repeated-measure ANOVA (group × time) revealed a highly significant interaction^h^. Thus, to increase the statistical power of *post hoc* comparisons of individual time bins the three groups of control mice were combined to form a single control group.

**Figure 3.**
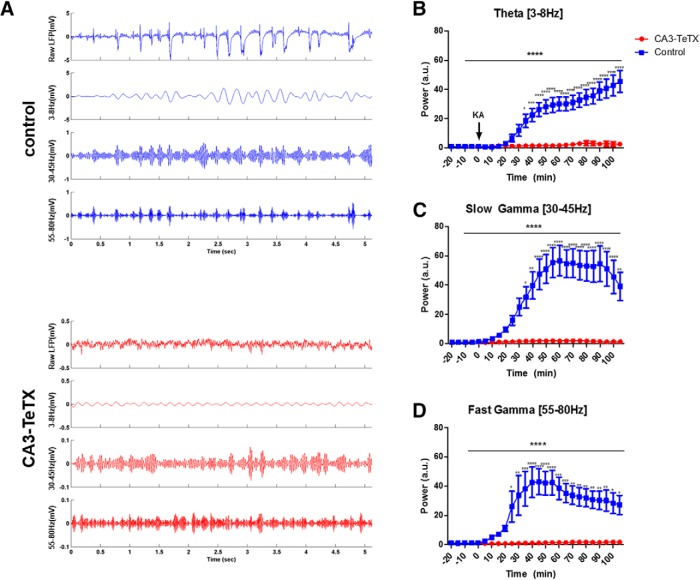
**Loss of CA3 output prevents increases in CA1 oscillations induced by KA. *A***, Example LFP recordings in CA1 stratum radiatum from a control (blue; top) and CA3-TeTX (red; bottom) mouse 60 min after kainic acid injection. For each genotype the top panel shows the raw LFP, the second panel the theta band filtered LFP, the third the slow gamma filtered LFP, and the bottom the fast gamma filtered LFP. Note the scale in the control traces is 10× that of the CA3-TeTX mice. KA injection lead to a significant increase in power in the (***B***) theta band and the (***C***) slow gamma band 35 min postinjection, and in the (***D***) fast gamma band 25 min following KA. ****p<0.0001, ***p<0.001, **p<0.01, *p<0.05. Control, *n*=22 (control ON DOX, *n*=12; control OFF DOX, *n*=4; CA3-TeTX ON DOX, *n*=6); CA3-TeTX, *n*=16.

In nearly all control mice, KA injection rapidly lead to an overall increase in the amplitude of the LFP, with the appearance of rhythmic epileptic discharges in the str. radiatum. However, in the majority of the CA3-TeTX mice the post-KA LFP was indistinguishable from the baseline period (Fig. 3*A*). Following KA injection normalized theta amplitude remained stable in the control mice for between 20 and 30 min and then began a steady increase over the next 30 min, at which time there was an obvious and significant difference compared with the CA3-TeTX mice, which showed no change from baseline conditions^i^ [two-way repeated-measure ANOVA, *F*_(25,900)_ (genotype × time) =17.86, *p*<0.0001, Bonferroni *post hoc* test, *p*<0.05 from 35 min post-KA; Fig. 3*A*,*B*]. The response to KA injection in both the slow and fast gamma frequencies was similar. Slow gamma power was significantly larger in the control mice 35 min following injection and remained elevated for the duration of the recording^j^ [two-way repeated-measure ANOVA, *F*_(25,900)_ (genotype × time)=9.69, *p*<0.0001, Bonferroni *post hoc* test, *p*<0.05 from 35 min post-KA; Fig. 3*A*,*C*], whereas high gamma was significantly increased 25 min after KA and remained high until the experiment was completed^k^ [two-way repeated-measure ANOVA, *F*_(25,900)_ (genotype × time)=5.884, *p*<0.0001, Bonferroni *post hoc* test, *p*<0.05 from 25 min post-KA; Fig. 3*A*,*D*].

## Discussion

Here we applied the kainic acid seizure model to CA3-TeTX mice, which lack CA3 pyramidal cell transmission. Our results validate the long-standing hypothesis that KA-mediated excitation of these cells is the key step in the ability of the drug to trigger seizure (Traub et al., 1996; Ben-Ari and Cossart, 2000; Vincent and Mulle, 2009). The CA3-TeTX mice have been a valuable tool in understanding the contribution of CA3 synaptic transmission to learning and memory (Nakashiba et al., 2008, 2009). Previous *in vitro* recordings have shown that the induction of TeTX expression leads to a drastic reduction in CA3 to CA1 transmission and a complete loss of CA3 generated population spikes in CA1, even at very strong stimulation intensities (Nakashiba et al., 2008). Although CA3–CA3 recurrent transmission has not been assessed physiologically in these mice, a loss of VAMP2 staining, an indicator of TeTX-mediated cleavage, is pronounced in the stratum radiatum and oriens of both CA1 and CA3 (terminals of CA3 pyramidal cell projections), as well as in the inner molecular layer of the DG, the location of the termination of projections from hilar mossy cells (MCs). However, no reduction is seen in terminals of the mossy fibers. These data suggest that CA3 axons projecting to both CA1 and CA3 are unable to release neurotransmitter. The loss of mossy cell transmission has not been directly investigated; however, the reduction of VAMP2 suggests this circuit may also be affected. Interestingly, a mouse lacking MCs has been shown to have increased excitability in the DG (Jinde et al., 2012) and a loss of MC transmission has been linked to temporal lobe epilepsy (Scharfman and Bernstein, 2015). We find it important to note that these phenotypes are the opposite of what we see in our current data, suggesting any effect on MC transmission in the CA3-TeTX mice should not impact the interpretation of our results. A further feature of the CA3-TeTX mice is the relatively slow time line of TeTX induction, on the order of 3 weeks. Although all our experiments were conducted soon after this time point, it is possible that adaptive changes in the hippocampal circuit may have occurred as a consequence of CA3 silencing. However, previous work found no cell death, nor obvious changes in presynaptic or postsynaptic markers in the hippocampus at this time point (Nakashiba et al., 2008), thus, we find it unlikely any compensatory changes are responsible for the resistance to KA seizure induction we observed. Despite the caveats outlined here, we feel our results strongly suggest that the loss of CA3 output prevents KA-induced seizures, limits the spread of neuronal excitation from the DG into the CA fields, and abolishes the ability of KA to generate synchronous oscillatory activity in the hippocampal network.

The repetitive limbic seizures that follow KA administration have been shown to be offset by interventions that augment inhibition (Halonen et al., 1995; Jinde et al., 2009). However, the intervention that resulted in the most profound resistance to KA-induced seizure to date has been the genetic deletion of the GluR6 kainate receptor subunit (Mulle et al., 1998). Our behavioral data is highly similar to the data obtained in the GluR6^−/−^ mice; a standard systemic dose of KA (20 mg/kg) failed to induce detectable seizures in virtually all of the Ca3-TeTX mice. Together these findings suggest the absolutely necessity of GluR6-mediated excitation of CA3 pyramidal cell in the generation of KA-induced seizure, assigning both a receptor and a network location to the initial step in the progression to status epilepticus.

On the level of neuronal excitation, KA can induce a strong depolarization of CA3 pyramidal cells, acting on GluR6-contained receptors located at the mossy fiber synapses (Robinson and Deadwyler, 1981). In our CA3-TeTX mice, we have prevented synaptic vesicle release from these neurons, without changing their intrinsic excitability. The lack of *c-fos* expression in CA3 of these mice following KA injection indicates that KA-mediated excitation alone, in the absence of activation of the CA3 recurrent collaterals, is insufficient to trigger activity induced gene expression. Further, we still observed robust *c-fos* expression in the granule cells of the DG in the CA3-TeTX mice, suggesting these neurons can respond to KA, possibly through a disinhibitory mechanism. However, it is interesting to note that KA failed to induce IEG expression in the DG granule cells of GluR6^−/−^ mice (Mulle et al., 1998), raising the possibility that this receptor is responsible for activation of IEG expression in these neurons in our model.

Increased and persistent oscillatory activity, particularly in the gamma bands, is a hallmark of both KA-induced pathophysiology and seizure onset in human patients (Uhlhaas and Singer, 2006). Inhibitory interneurons in all hippocampal subfields are known to be directly excited by KA via the GluR5 receptors (Fisahn et al., 2004) leading some to suggest that KA-induced gamma oscillations could emerge as a result of excitation of these neurons alone (Whittington et al., 1995; Craig and McBain, 2015). However, our data demonstrate that excitatory transmission from CA3 PCs is absolutely necessary for this network activity *in vivo.* CA3 is known to be key in the generation of gamma oscillations both *in vivo* and *in vitro*, thus, it is perhaps not surprising that silencing of this network prevents KA-induced gamma in our experiments. Nonetheless, these data clearly define the necessity of this circuit in generating population oscillations. This is in contrast to the surprising observation that mice lacking NMDA receptors (NRs) specifically in CA3 pyramidal cells were hypersensitive to KA-induced seizures, despite being unable to generate KA-induced gamma oscillations in CA1 (Jinde et al., 2009). In that study, the authors hypothesized that the loss of NRs in CA3 limited the ability of CA3 to trigger GABA release in CA1 following KA. Further, they showed that pretreatment with an enhancer of presynaptic GABA release rescued the KA-induced gamma oscillations in the mutants and reduced epileptiform activity. In our mutant mice, we see a parallel in the loss of gamma oscillations and seizure activity following KA, again suggesting a reduction in inhibition alone is insufficient for either consequence of KA treatment.

Although this current study establishes the necessity of CA3 transmission in this model system, it does not address whether seizures could be triggered in these mice with higher doses of KA or other drugs that target CA3, such as carbachol, 4-AP, or bicuculline. This is of importance, as although we observed reliable seizure induction in our control groups (Fig. 1), the background strain of our mice, C57BL/6, has been suggested to be resistant to the excitotoxic effect of KA (Schauwecker and Steward, 1997). Further, increasing the dose of KA to 30 mg/kg was sufficient to induce seizure in GluR6^−/−^ mice (Mulle et al., 1998), suggesting at higher concentrations alternate circuits, perhaps involving the amygdala, may be activated and/or KA can act on other receptor types, such as AMPA receptors (Chergui et al., 2000; Tomita et al., 2007). Additionally, here we only examined the acute effects of KA administration; it would be of interest in future work to examine the long-term effects of KA on cell death in CA1 and chronic changes in hippocampal physiology.

Although it had been suggested that KA-mediated depression of inhibitory transmission in CA1 could play a crucial role in epileptiform activity, a “collapse of inhibition” model (Fisher and Alger, 1984; Clarke et al., 1997; Rodríguez-Moreno and Lerma, 1998), our results suggest this action of the drug alone is insufficient to induce seizure. Rather, our data strongly align with the more contemporary view that KA generates excitation of CA3 pyramidal cells while simultaneously strongly increasing tonic inhibition across the circuit (Ben-Ari and Cossart, 2000). Whereas in normal animals this increased inhibition does not protect the network from seizure induction because of the strong excitation of CA3 pyramidal cells, first directly by the drug, then amplified by the strong CA3 recurrent collaterals, in our mutants the lack of CA3 transmission preserves a strongly inhibitory tone, blocking both seizure induction and hippocampal synchrony.

## Synthesis

The decision was a result of the Reviewing Editor Uwe Heinemann and the peer reviewers coming together and discussing their recommendations until a consensus was reached. A fact-based synthesis statement explaining their decision and outlining what is needed to prepare a revision is listed below. The following reviewers agreed to reveal their identity: Heinz Beck, Gudrun Ahnert-Hilger

In the presented paper the authors describe the impact of silencing CA3 principal neurons on the development of kainate induced seizures. For specific neuronal silencing they used the dox-dependent Cre Tetx approach by which CreTeTx genetically changed mice are used. TeTx light chain expression is hindered during postnatal development by feeding the animals with doxycycline. Expression is induced by changing to normal diet at the desired time point. This elegant approach gives an individually triggered start of the Tetx LC expression and inhibition of neurotransmission in the respective neurons. In treated animals, the authors could show that following kainate injection the epileptic spread to CA1 depends on neurons of the CA3 area.

The experiments are well performed and convincing. The number of animals for each data set should be included in the figure legend. The English needs some work.

The second reviewer writes that the value of this study is the prospective validation of the hypothesis that CA3 is critically involved in generation of kainate seizures. The authors present evidence showing that silencing CA3 output i) attenuates behavioral seizures, ii) attenuates c-fos staining increases after kainate injection in CA3 and downstream CA1, but not in dentate gyrus, iii) attenuates CA1 kainate-induced activity measured with silicon probes. I have no large issues with these results themselves. However, I do think it would be good to acknowledge some limitations of the model, or to clarify that they are irrelevant. There are a few other minor points that will likely be easy to remedy.

- The authors have used the triple transgenic mice for repression of synaptic transmission as far as I can see as established in Nakashiba et al. 2008. In this paper , the authors claim selectivity to CA3, but the supplementary Fig. S1 shows expression of GFP not only in CA3, but also in the superficial cell layers of the dentate gyrus. This might also be fibers in the IML, it is really hard to be sure at the scale provided. I think it would be nice to evaluate in the mice the authors used if the selectivity of expression is indeed only CA3, or if it is more extensive.

- Secondly, the physiological evidence in this inducible TeNT approach is so far to my knowledge confined to the recording of CA3- CA3 projections using field potentials in Nakashiba et al. 2008. I think it is worth mentioning that i) this was not complete silencing, ii) that frequency stimulation was not tested (i.e. facilitation), and iii) that the function of the recurrent CA3 network was not investigated. This would require some rewording to make clear that what the authors can prove so far is that inducible reduction of the CA3 to CA1 output (but not the CA1 temporoammonic inputs for instance) causes strong changes in CA1 activity and seizures. It is unclear what happens to CA3-CA3, even if it is quite probable that these synapses are also silenced.

- Thirdly, the authors should comment on the possibility of adaptive changes in the 20+ days needed for TeNT expression. Unlikely to explain the results, but I think it should be addressed.

In addition, there is one statistical point. I am not really convinced by the procedure of combining all three types of control mice because there was no difference between them into a single control group. Of course I realize that there are advantages power-wise to doing this. However, there are perfectly fine ways of testing differences between 4 groups with ANOVA and appropriate post tests. I think this should be done and reported, if the authors wish together with their combined-group statistics. In this way, the reader can form his own opinion on how the variance between the control groups affects the results.

Finally, I think the authors have to tone down the rather bald statement at the beginning of the discussion that essentially says that seizures induced by kainate are similar to human epilepsy, and that this has made the model valuable to understand epileptogenesis. This is mixing two issues, ictogenesis and epileptogenesis. The first issue, ictogenesis: It is to my mind not clear at all if human temporal lobe seizures mirror kainate-induced seizures (apart from the obvious commonalities between all seizures). Epileptogenesis is not a topic here, and the similarities in ictogeneic mechanism have certainly not made this a useful epileptogenesis model. Rather, kainate mice or rats make for a pretty good phenocopy of temporal lobe epilepsy with Ammon's horn sclerosis (beside the point for this paper).

Finally I would like the authors to shortly comment whether the light chain toxin can be released from dying cells and then incorporated into other cells thereby modifying the network.
